# Supplementary figures and images for: Lysicamine Reduces Protein Kinase B (AKT) Activation and Promotes Necrosis in Anaplastic Thyroid Cancer
Source: Pharmaceuticals (Basel). 2023 Dec 4;16(12):1687. doi: 10.3390/ph16121687 (PMC10748177; doi:10.3390/ph16121687)

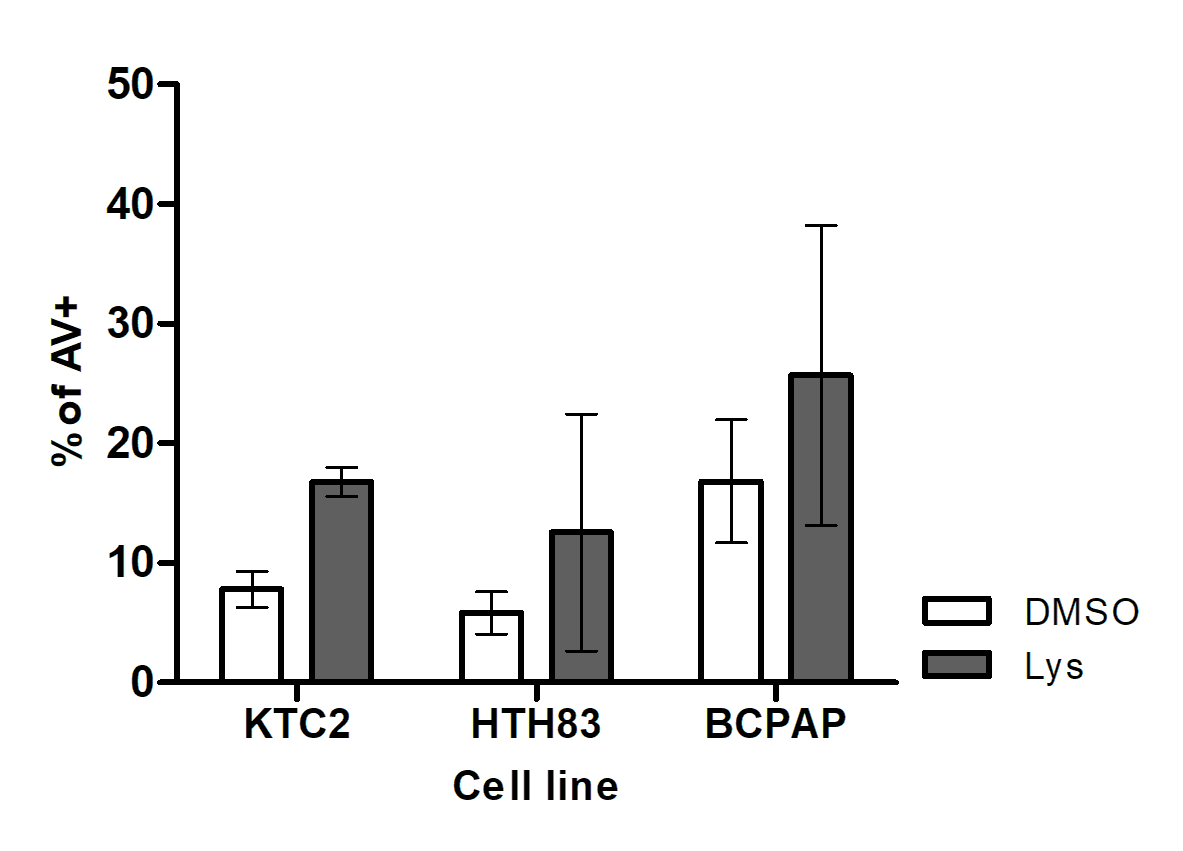

Supplement: Supplementary file 1 [file pharmaceuticals-16-01687-s001.zip › pharmaceuticals-2665721-Figure S1.tif]
